# Food Authentication: Techniques, Trends and Emerging Approaches

**DOI:** 10.3390/foods9030346

**Published:** 2020-03-17

**Authors:** Raúl González-Domínguez

**Affiliations:** 1 Department of Chemistry, Faculty of Experimental Sciences, University of Huelva, 21007 Huelva, Spain; raul.gonzalez@dqcm.uhu.es; Tel.: +34-959-219-975; 2 International Campus of Excellence CeiA3, University of Huelva, 21007 Huelva, Spain

Multiple factors can directly influence the chemical composition of foods and, consequently, their organoleptic, nutritional and bioactive properties, including the geographical origin, the variety or breed, as well as the conditions of cultivation, breeding and/or feeding, among others. Therefore, there is a great interest in the development of accurate, robust and high-throughput analytical methods to guarantee the authenticity and traceability of foods. For these purposes, a large number of sensorial, physical and chemical approaches can be used, which must normally be combined with advanced statistical tools. In this vein, the aim of the Special Issue “Food Authentication: Techniques, Trends and Emerging Approaches” was to gather original research papers and review articles focused on the development and application of analytical techniques and emerging approaches in food authentication. This Special Issue is comprised of 12 valuable scientific contributions, including one review article and 11 original research works, dealing with the authentication of foods with great commercial value, such as olive oil, Iberian ham or fruits, among others.

Morisasa et al. reviewed the potential of matrix-assisted laser desorption/ionization mass spectrometry (MALDI-MS) imaging as a valuable technique to determine small metabolites in food tissue sections without requiring purification, extraction, separation or labeling processes [1]. They highlight that MALDI-MS can be employed not only to identify the nutritional content of foods, but also to investigate their geographical origin for improved traceability, food safety and breed enhancement, among other applications. However, the authors also emphasize that further technical improvements are needed, especially to overcome sensitivity issues.

Several research articles reported the application of chromatographic-based analytical approaches for profiling different analytes as possible chemical descriptors for authenticity and traceability purposes. Rueda et al. determined the free amino acid content of Iberian dry-cured hams to differentiate among three ripening stages: postsalting, drying and cellar [2]. For this purpose, they employed gas chromatography coupled to mass spectrometry (GC-MS) and flame ionization detector (GC-FID) to identify and quantify 18 amino acids. Alanine, tyrosine, glutamine, proline, 2-aminobutanoic acid, cysteine and valine were found to be the most differentiating amino acids between the ripening stages by using principal component analysis (PCA) and linear discriminant analysis (LDA), which could be therefore used to predict the curing time. Volatile profiling by GC-MS (alcohols, aldehydes, hydrocarbons, terpenoids) combined with mineral content determination (25 macro- and microminerals) was employed to classify prickly pear juice samples from the Peloponnese Peninsula according to the geographical origin [3]. Multivariate analysis demonstrated that seven minerals and 21 volatile compounds provide satisfactory classification rates, and furthermore, mineral content of soil samples was satisfactorily correlated with mineral levels detected in fruit juices. Similarly, Papapetros et al. also investigated volatile and mineral profiles, combined with other conventional physicochemical and spectroscopic determinations (e.g., acidity, total phenolic content, sugars), to differentiate sweet cherry samples grown in northern Greece according to the botanical origin [4]. Results evidenced that individual datasets provide acceptable but not satisfactory classification rates, whereas their combination leads to improved classification models. In another study, two Spanish Protected Designation of Origin vinegar samples were analyzed for polyphenol and volatile content by liquid and gas chromatography approaches, respectively [5]. Multivariate data analysis demonstrated clear differences between vinegars with regard to their polyphenolic content, and to a lesser extent, in the volatile fraction. Authors proposed that these differences should be mainly due to varietal and geographical factors, since vinegar manufacturing and ageing processes are similar in both regions. To achieve a comprehensive characterization of the chemical composition of strawberry fruits, González-Domínguez et al. applied a multitargeted profiling approach to determine multiple compounds related to sensory and health characteristics of this berry fruit, including sugars, organic acids, polyphenols and mineral elements [6]. Then, several complementary pattern recognition procedures were employed to discriminate strawberry varieties grown under different climatic and agronomic conditions. Anthocyanins, phenolic acids, sucrose and malic acid showed significant differences among cultivars, while climatic conditions and the cultivation system were responsible for changes in polyphenol contents. In this vein, metabolomics has also been proposed as a powerful screening tool for authenticity assessment [7]. Targeted and nontargeted metabolomics approaches were used to detect pomegranate juice adulteration with apple and red grape juice. This methodology allowed distinguishing adulteration to levels below 1%, and 80 potential biomarkers were identified (e.g., anthocyanins, flavonoids).

The use of spectroscopic methods for food authenticity research has also been reported in some research articles published in this Special Issue, as detailed below. Campmajó et al. described the application of high-performance liquid chromatography with ultraviolet detection (HPLC-UV) to detect “fingerprints” for the classification of hen eggs according to their production method: organic, free-range, barn or caged [8]. Multivariate modeling enabled satisfactory discrimination rates, especially for the distinction among organic and nonorganic eggs. However, perfect classification of the four egg groups was not achieved, so authors proposed that future research lines could include the evaluation of egg yolk instead of the whole egg, and the use of fluorescence detection as a more selective technique. Using a similar analytical approach based on LC-UV fingerprinting, Bikrani et al. were able to differentiate margarines and fat-spread-related products from different geographical origins from Spain and Morocco [9]. Several multivariate chemometrics tools were compared, with partial least squares-discriminant analysis (PLS-DA) being the statistical strategy that provided the best performance. In this line, luminescence also demonstrated a great potential to characterize edible oils and detect adulterations in a rapid way [10]. In this work, a regression model based on five luminescent frequencies, associated with minor oil components, was designed and validated for detecting virgin olive oil adulteration with hazelnut oil.

Piarulli et al. developed a robust DNA-isolation protocol from extra virgin olive oil (EVOO) for subsequent polymerase chain reaction (PCR)-based fingerprinting [11]. This method was then successfully applied for genetic tagging of filtered EVOOs of unknown origin. Finally, the work by Minnens et al. aimed to investigate attitudes towards a food integrity information sharing system (FI-ISS) among stakeholders in the European food supply chain [12].

In summary, the Special Issue “Food Authentication: Techniques, Trends and Emerging Approaches” evidences the great importance of developing novel analytical approaches to define accurate and reproducible indicators for food authenticity and traceability. At the same time, as suggested by several authors, the application of advanced chemometrics approaches is also essential to achieve robust results, with the aim of characterizing food composition, discovering potential markers (e.g., adulteration) and obtaining satisfactory classification models.
